# A MEMS Electrochemical Seismometer Based on the Integrated Structure of Centrosymmetric Four Electrodes

**DOI:** 10.3390/mi13030354

**Published:** 2022-02-23

**Authors:** Yumo Duan, Anxiang Zhong, Yulan Lu, Jian Chen, Deyong Chen, Junbo Wang

**Affiliations:** 1 State Key Laboratory of Transducer Technology, Aerospace Information Research Institute, Chinese Academy of Sciences, Beijing 100190, China; duanyumo19@mails.ucas.ac.cn (Y.D.); zhonganxiang19@mails.ucas.ac.cn (A.Z.); luyl@aircas.ac.cn (Y.L.); chenjian@mail.ie.ac.cn (J.C.); 2 School of Electronic, Electrical and Communication Engineering, University of Chinese Academy of Sciences, Beijing 100049, China

**Keywords:** electrochemical seismometer, MEMS, centrosymmetric four electrodes

## Abstract

This paper presented an electrochemical seismic micro sensor based on an integrated structure of four centrosymmetric electrodes. In this integrative structure, cathodes were not only distributed on wafer surfaces but also on the inner walls of the flow holes of the wafer, which increased the effective cathode areas and improved the sensitivity of the sensor. Numerical simulations were conducted to validate the feasibility of the integrated structure of four centrosymmetric electrodes in monitoring seismic vibrations where variations in the arrangements of the flow holes and anode width were investigated. The integrated structure of the four centrosymmetric micro electrodes was fabricated based on Micro-Electro-Mechanical Systems (MEMS) without the requirement of manual alignments. Experimental characterizations revealed that: (1) the maximum sensitivity of the electrochemical seismic sensor based on the integrated structure of four centrosymmetric electrodes was two orders of magnitude higher than that of the commercial counterpart of CME6011 and three times higher than the electrochemical seismic sensor based on the integrated structure of four planar micro electrodes; (2) the electrochemical seismic sensor based on the integrated structure of four centrosymmetric micro electrodes demonstrated comparable and even lower noise levels in comparison to CME6011. Thus, the electrochemical seismic micro sensor developed in this study may function as an enabling tool in future applications of seismic monitoring and geophysical explorations.

## 1. Introduction

Seismic sensors which have key applications in seismic monitoring and geophysical exploration are sensors that convert signals of ground vibrations as mechanical energies into electrical signals or energies for analysis [1,2,3,4]. Traditional seismic sensors can be divided into moving coil seismic sensors [5,6], optical fiber seismic sensors [7,8], capacitive seismic sensors [9,10], piezoelectric seismic sensors [11], and electrochemical seismic sensors. Compared with other types of seismic sensors, electrochemical seismic sensors have outstanding advantages. Firstly, an electrolyte coupled with an elastic membrane is used as an inertial mass without the requirements of adjusting positions and centering so that the working inclination of the electrochemical seismic sensor is large. Secondly, there are no precision and moving mechanical parts included, ensuring that the sensor also has the advantages of simple operations and high impact resistance. When active ions move in flow channels under low-frequency seismic vibrations, they have enough time to diffuse to electrode surfaces, producing high responses at the low-frequency domain for the electrochemical seismic sensor. Therefore, compared with other types of seismometers, electrochemical seismometers are more suitable for seabed seismic monitoring in a complex underwater environment [12].

Early electrochemical seismic sensors used meshes of platinum wires as electrodes and ceramic flakes or polymers as insulating layers, which were then assembled by conventional approaches of ceramic sintering. This traditional process has many disadvantages, such as poor consistency, complex processes, and high cost [13,14]. Then MEMS technologies were introduced into the fabrication of electrochemical seismic sensors. He et al. assembled several wafers as electrodes and insulating layers to form electrochemical seismic micro sensors, which were very difficult to align properly during assembly [15]. Then, in order to obtain high sensitivity, Deng et al. developed a sensing unit [16,17] that integrated layers of electrode and insulation on the same silicon wafer, and then even integrated a pair of anodes and cathodes on a single chip. Although it reduced the difficulty of alignments, manual alignment was still an essential step, which severely affected the consistency of sensors and, also, could lead to sensor damages.

Xu used a round-hole-network structure to integrate an anode and cathode pair on a wafer and then assembled two wafers and O-shaped rubber rings into a sensitive unit. This structure not only increased the cathode areas by distributing cathodes on the side walls of the flow holes but also made the anodes evenly distributed near the flow holes, which greatly improved the output sensitivities of the sensor. However, the two wafers still needed to be manually aligned and assembled [18]. In a subsequent study, Xu proposed the integrated electrodes, which employed a three-layer anodic bonding structure of silicon-glass-silicon, served as the substitutes for multilayer manual assembly structures [19]. However, the process was complex, and the yield was low.

In order to completely solve the problem of manual alignments, Zheng developed an electrochemical seismic sensor, integrating two pairs of anode and cathode electrodes on a single wafer [20]. However, due to problems in structural design, cathode electrodes were only distributed on wafer surfaces, and the cathode in the center of the wafer was difficult to use, resulting in compromised cathode areas and low sensitivities.

In this paper, an integrative structure with central symmetrical micro electrodes was developed, which was improved on the basis of the round-hole-network structure. The traditional axisymmetric distribution of electrodes was changed to a centrosymmetric distribution, and through holes perpendicular to electrode surfaces were arranged in parallel as flow channels of electrolytes. Firstly, the network distribution of the anode and cathode pair and the cathode on the inner wall of the flow hole inherited its advantages of high sensitivity. Secondly, the four electrodes were integrated on the same wafer at the same time to facilitate easy assembly and improve device consistency.

## 2. Structure and Working Principle

**Figure 1:**
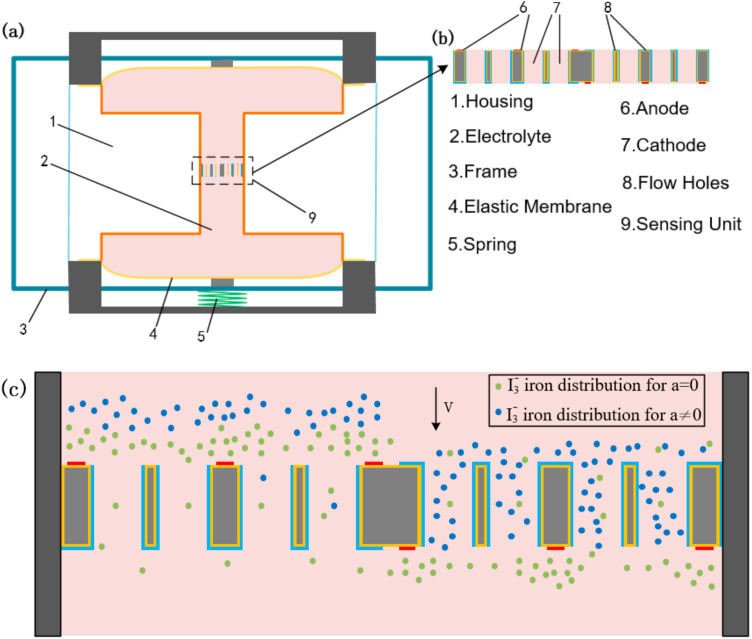
(**a**,**b**) Structure of the MEMS-based electrochemical seismometer which was mainly composed of an integrative structure with four centrosymmetric electrodes; (**c**) The working principle of the seismometer which characterized the outside vibration by the concentration gradient of ${\;I}_{3}^{-}$.

As shown in Figure 1, the electrochemical seismic sensor was mainly composed of the following parts: a frame, two elastic membranes, a spring, and a main channel filled with an electrolyte solution. The electrolyte solution was a mixed solution of I_2_ and KI, in which the concentration of KI was 50–100 times that of I_2_. The sensing unit of the sensor was placed in the center of the main channel, which was encapsulated with the electrolyte in a plexiglass shell sealed by two elastic membranes. As shown in this figure, the sensing unit was the core of the sensor, including two pairs of cathodes and anodes, integrated symmetrically about the center of the same wafer, and through holes perpendicular to the electrode surface arranged in a certain way as a flow channel for electrolyte flow. The cathode was not only distributed on the electrode surface but also on the inner walls of the flow holes, which increased the effective cathode areas and improved the sensitivity of the sensor. Integration of the four electrodes on the same wafer reduced the flow resistance to simplify the installation process without manual alignments to improve the consistency of the sensor.

When a stable DC voltage (0.3 V in this study) was applied to the two pairs of electrodes of the sensor, a reversible electrochemical reaction occurred around the anode and cathode, as shown in Formula (1):

(1) $$I^{-}+I_{2}⇌I_{3}^{-}$$

After the voltage was applied for a period of time, positive and negative reactions reached an equilibrium, and a stable starting current was maintained in the solution and the external circuit. When there was no external vibration, the ion concentration distribution around the two pairs of electrodes was symmetrical, and the output current was the difference between the two cathode currents equal to 0. When external vibration occurred, the electrolytes flowed relative to the sensing unit, and the velocity field could be described by the Navier–Stokes equation of incompressible fluid [21].

(2) ∇·u ⃗=0

(3) $$\rho_{e}d\;\vec{u}/\mathrm{dt}=-\nabla p+\mu \nabla^{2}\;\vec{u}+\;\vec{a}$$

where $\;\vec{u}$ is the velocity vector, $\rho_{e}$ is the electrolyte density, t is the time, p is the pressure, μ is the dynamic viscosity, and a is the acceleration vector. With the flow of electrolytes, the distribution of $I_{3}^{-}$ ion concentration also changed because of inertia. The Nernst–Planck equation, under the assumption of electrical neutrality, could be used to solve the problem of ion transports:

(4) $$\;\vec{N}=\;\vec{u}C-D\nabla C-\mathrm{zmFC}\nabla \varphi$$

where N is $I_{3}^{-}$ ion flux, C is the solution concentration, z is the charge, D is the diffusion coefficient, $9.6485\times 10^{4}C/\mathrm{mol}$ is the Faraday constant, φ is the potential difference, the electron mobility m=D/RT, and R is the gas constant. Three terms at the right end corresponded to the changes of ion flux caused by convection, diffusion, and electromigration. The convective flux in the main channel was mainly caused by external vibration, which could be calculated by Equation (2). Since there were a large number of $I^{-}$ ions in the solution, its shielding effect ignored the influence of electromigration, so diffusion played a major role. At the same time, there was no flux at the boundary of channels. According to the characteristics of electrode reaction, the Bulter–Volmer equation related to ion flux could be established at the electrode boundary to represent the reaction ion flux [22]:

(5) $$\begin{array}{c} 2\vec{n}\cdot {\;\vec{N}}_{I_{3}^{-}}=-\frac{2\vec{n}\cdot {\;\vec{N}}_{I^{-}}}{3}=-k_{a}2C_{I_{3}^{-}}e^{\left(-\alpha \mathrm{nF}/\mathrm{RT}\right)\left(U-E_{0}-\varphi \right)}+\\ k_{c}C_{I^{-}}e^{\left(1-\alpha \right)\left(\mathrm{nF}/\mathrm{RT}\right)\left(U-E_{0}-\varphi \right)} \end{array}$$

where n is the unit normal vector outward from the electrode surface, $k_{a}$ and $k_{c}$ are the reaction constants of anode and cathode, respectively, N is the number of electron exchanges in the reaction (in this formula, n = 1), α is the charge transfer coefficient, u is the DC voltage applied on the electrode, and $E_{0}$ is the equilibrium potential. The relationship between the cathode current and reaction ion flux can be expressed as follows:

(6) $$I=\mathrm{nF}\int_{S}^{\;}\;\vec{n}\cdot {\;\vec{N}}_{I_{3}^{-}}\mathrm{dS}$$

where S is the cathode surface area. It can be seen from the above formula that when the electrolytes flowed, the different $I_{3}^{-}$ ion concentration distribution around the two cathodes led to different cathode current intensities. The output of the final sensor is as follows:

(7) $$U_{0}=\left(I_{1}-I_{2}\right)\times R$$

where $U_{0}$ is the differential output voltage,${\;I}_{1}$, $I_{2}$ are the output currents of two cathodes, respectively, and R is the conversion resistance. In this way, the external vibration could be converted into the output of the sensor.

## 3. Simulation

Due to the complex principle of the electrochemical seismic sensor, finite element simulation (COMSOL Multiphysics 5.5, Stockholm, Sweden) was selected to divide the sensing process into a vibration link and an electrochemical link. The feasibility of the sensor was verified, and two parameters (e.g., arrangement of flow holes and anode width) affecting the output sensitivity of the sensor were selected for analysis and optimization.

**Figure 2:**
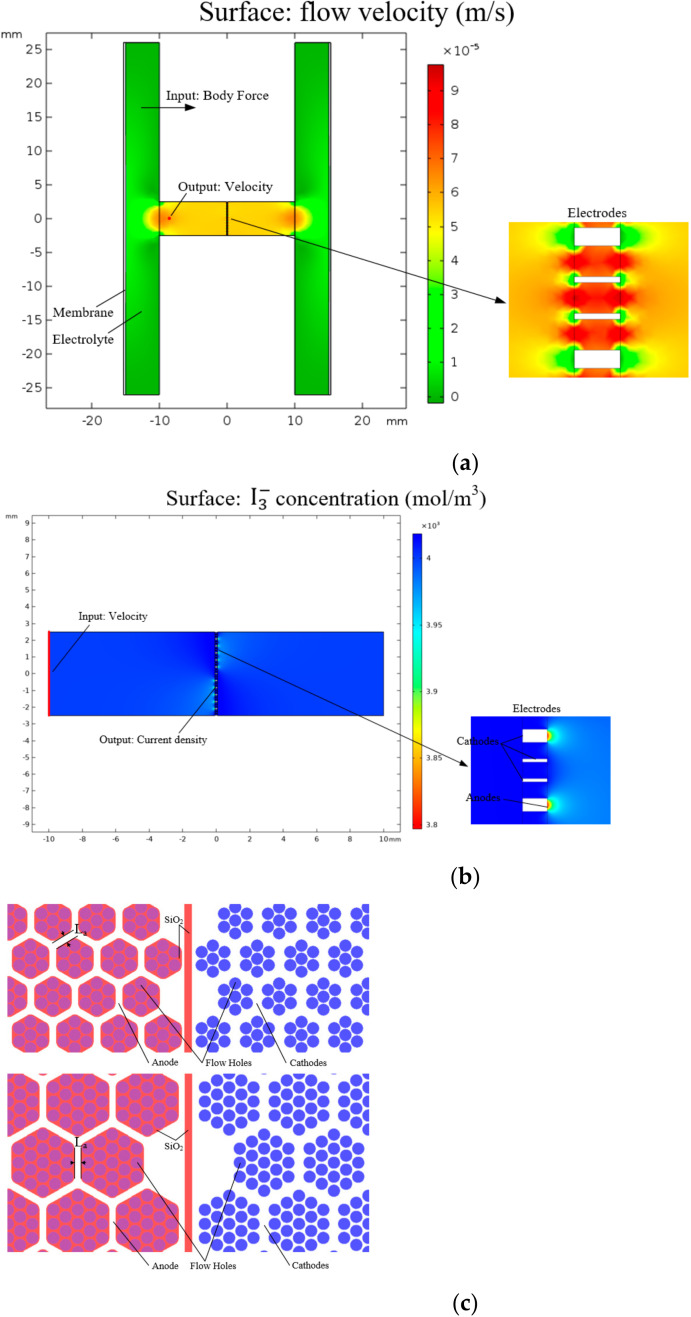
Simulation models: (**a**) Simulation model of the vibration link which took the volume force as the input and the liquid velocity at the midpoint of the channel edge as the output. (**b**) Simulation model of the electrochemical link which took the linear velocity of the end face of the channel as the input and the differential current of the two cathodes as the output. (**c**) Two arrangements of flow holes with the seven-hole structure on the top and the nineteen-hole structure at the bottom.

In the simulation, the continuity equation and Navier–Stokes equation were used to solve the velocity distribution of electrolytes in the channel. Faraday’s law, Butler–Volmer condition, and Nernst’s plan equation were used to solve the ion concentration distribution. The vibration link was the process of converting an external vibration into low rates of the electrolyte with the simulation model shown in Figure 2a. In the simulation of this link, the “fluid–solid coupling” physical field was used, with the volume force applied to the electrolyte as the input and the liquid flow rate at the midpoint of the edge of the flow channel as the output. The electrochemical link was the process of converting the electrolyte flow rate into the output of the sensor with the simulation model shown in Figure 2b. The physical fields of “laminar flow” and “tertiary current distribution” were used in the simulation of this link, with the linear velocity of the fluid at the end of the channel as the input and the differential current of two cathodes as the output. In the simulation, the electrolyte flow was regarded as incompressible laminar flow at room temperature, the solid walls were in non-slip condition, the effect of electromigration was ignored, and the mesh of the model was divided by physical field control mesh. The final transfer function of the sensor was the multiplication and addition of the transfer functions of two links.

**Figure 3:**
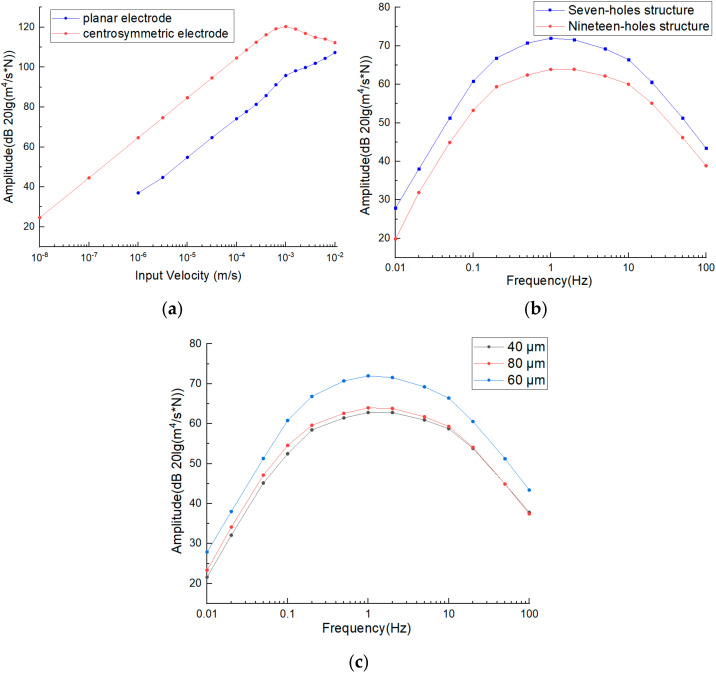
Simulation results: (**a**) output current vs. input velocity of the electrochemical seismic sensors based on the integrated structure of four centrosymmetric micro electrodes vs. four planar micro electrodes. Amplitude frequency responses of the electrochemical seismic sensors based on the integrated structure of four centrosymmetric micro electrodes with variations in arrangements of flow holes (**b**) and anode width (**c**).

Firstly, the feasibility of structural design was verified by simulation. Since the electrode structure adopted the design of central symmetry, the anode of one pair of electrodes and the cathode of the other pair of electrodes were on the same side of the wafer. It was necessary to verify whether the anode electrode could affect the output current of the cathode on the same side, making it difficult for the sensitive unit to output differential currents normally. The simulation was designed as follows. The electrochemical link was simulated where the external vibration frequency was fixed at 1 Hz, and the input speed was changed to verify whether the differential current output of cathodes was normal. At the same time, the integrated structure with four planar electrodes [19] was selected for comparison. The simulation results are shown in Figure 3a.

The results showed that differential currents of central symmetrical cathodes increased linearly with the increase in electrolyte flow rates, which verified the feasibility of the device. In addition, it can be seen from the simulation that compared with the integrated structure of the four planar electrodes, the structure with central symmetrical electrodes made full use of the areas on the wafer surface and inner walls of the flow channels. When the distance between cathodes and cathodes was decreased, the cathode could increasingly make full use of the charged ions generated by the anode, improving the output sensitivity of the electrochemical link. It can be seen that the output sensitivity of the electrochemical link of the device decreased when the external input was large, which may be due to the fact that the charged ions generated by the electrochemical reaction of the anode could not react with the cathodes in time when the flow rate was too high. In conclusion, the structure design of the central symmetrical electrodes was feasible and had a high sensitivity, which could effectively detect small signals.

Secondly, the influence of the pattern design on the sensitivity was analyzed. Due to process limitations and structural optimization, two arrangements of flow holes, as shown in Figure 2c, were designed. Seven holes or nineteen holes were used to form a unit according to the principle of dense stacking and then arranged according to a certain spacing (hereinafter referred to as seven-hole structure and nineteen-hole structure). Compared with the seven-hole structure, the nineteen-hole structure included more flow holes on one wafer, which was conducive to reducing the flow resistance and improving the performance of the vibration link. However, the anode area corresponding to each flow hole was small, and the hole utilization rate in the middle of the unit was low. By selecting the seven-hole and nineteen-hole structures with an anode width of 60 microns, the vibration link and electrochemical link was simulated. The second key parameter was the anode width L. As indicated in Figure 2c, as the anode width was increased, an increase in the electrochemical reaction abilities was found. However, further increase in anode width reduced the number of flow holes and increased the flow resistance, which was unfavorable to the vibration link. The seven-hole structures with anode widths of 40, 60, and 80 μm were selected for simulation. The simulation results are shown in Figure 3b,c. The results show that with the increase in input volume force frequency, the output amplitude first increases and then decreases. This is because the conversion efficiency between external vibration and electrolyte movement was relatively low in the low-frequency domain, and the electrochemical reaction rate on the electrode was limited in the high-frequency domain. From Figure 3b, it can be seen that under the condition of the same anode width, the output sensitivity of the seven-hole structure was higher than that of the nineteen-hole structure. It was found from the $I_{3}^{-}$ ion concentration distribution that most of the $I_{3}^{-}$ ions produced by the anode would enter the flow hole near the anode and react with the cathode, so the improvement of the vibration link caused by the reduction in the flow resistance was not enough to offset the influence on the electrochemical link caused by the deduction of the reaction efficiency. Thus, the seven-hole structure was a better choice. From Figure 3c, it was observed that under the same flow hole distribution, the anode width of 60 μm had the highest output sensitivity. It showed that this selection could well balance the influence of flow resistance and electrochemical reaction efficiency caused by the change of anode width and, thus, 60 μm was determined as the anode width.

## 4. Fabrication

**Figure 4:**
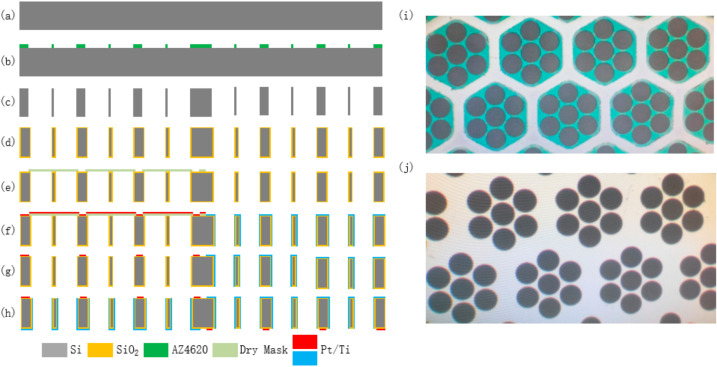
(**a**–**h**) Fabrication process of the integrative structure with four centrosymmetric micro electrodes. (**i**,**j**) Microscopic observation of flow holes on anode and cathode.

Figure 4 shows the fabrication process of the electrochemical seismic sensor, including thermal oxidation, lithography, metal sputtering, deep reactive ion etching, etc. In order to increase the electrode area as much as possible and improve the efficiency of electrochemical reactions between anodes and cathodes, the insulation interval between anodes and cathodes on the structure surface was 10 μm. In order to ensure the success rate of the etching process and increase the number of flow holes as much as possible, the thickness of the side walls between the flow holes was 20 μm. In addition, according to simulation results, the seven-hole structure was selected for the flow hole distribution, and the anode width was 60 μm.

(a) A silicon wafer with a thickness of 200 μm was selected and thoroughly washed in boiling sulfuric acid and deionized water; (b) a mask of patterned AZ4620 photoresist for etching was formed on one side of the silicon wafer by photolithography; (c) the patterned silicon wafer was etched by deep reactive ion etching (DRIE) to form flow holes (Chamber pressure: 10 Pa; Gas: SF_6_ 450 sccm, C_4_F_8_ 150 sccm; Power: Upper electrode 2500 W, Lower electrode 1500 W; Cycle: 170), and then the silicon wafer was cleaned with acetone, alcohol solutions, and deionized water to remove residual photoresist and other impurities; (d) a 1 μm-thick uniform silica insulating layer was formed on the surface of the silicon wafer by thermal oxidation (under 1080 °C); (e) a patterned dry film mask was formed on one side of the silicon wafer by lithography to transfer the electrode pattern to the silicon wafer; (f) on this side, 400 angstroms-thick Ti and 2500 angstroms-thick PT were sputtered successively (Chamber pressure: 6 × 10^−6^ Torr; Gas: Ar 35.5 sccm); (g) the anode of one pair of electrodes and the cathode of the other pair of electrodes were prepared simultaneously by lift-off; (h) repeated steps (e–g) on the other side of the silicon wafer to prepare the remaining two electrodes.

**Figure 5:**
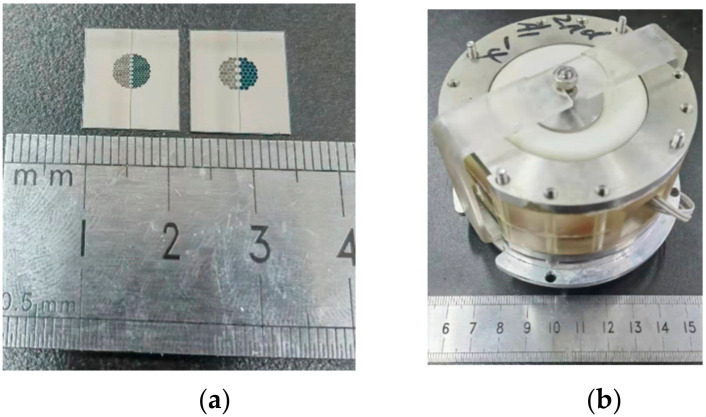
Prototyping images of the fabricated integrative structure of four centrosymmetric micro electrodes (**a**) which was assembled to form an electrochemical seismic micro sensor (**b**).

Figure 5a shows the sensing electrode prepared after cutting the silicon wafer, with the size of 12.3 mm × 14.3 mm. Then, four electrodes of the sensing unit were bonded with the lead, sealed, and packaged with an organic plastic shell and a rubber film. Then, an electrolyte solution with 0.02 mol/L iodine and 2 mol/L potassium iodide was injected.

## 5. Result

**Figure 6:**
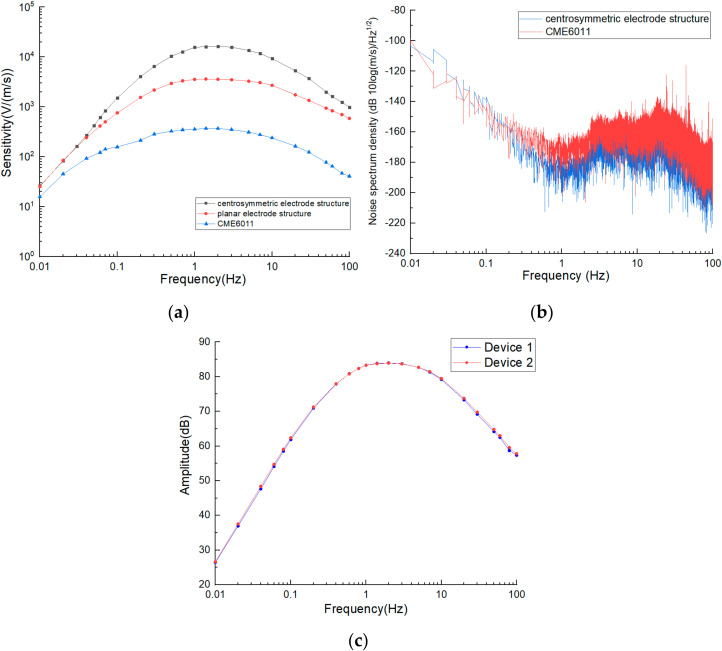
Characterization results: (**a**) amplitude frequency response and (**b**) noise spectrum densities of the electrochemical seismic sensor based on the integrated structure of four centrosymmetric micro electrodes, in comparison to the commercial counterpart of CME6011 and the electrochemical seismic sensor based on the integrative structure of four planar micro electrodes. (**c**) amplitude frequency response of two electrochemical seismic sensors based on the integrated structure of four centrosymmetric micro electrodes.

In order to test the performance of the electrochemical seismic sensor with four-electrode integration in this study, the commercial electrochemical seismic sensor CME6011 and the electrochemical seismic sensor with the planar four-electrode integrated structure [19] were selected for comparison. The seismic sensor was placed on a shaking table where the sine wave with predefined frequencies was generated by a signal generator and amplified by a power amplifier to create the vibration signal. The output current of the seismic sensor was converted into a voltage through a 1 kΩ conversion resistor. Figure 6a shows the amplitude frequency responses of three types of seismic sensors where the sensitivities increased with the increase in frequency at the low-frequency domain. After reaching the maximum sensitivities, the sensitivities decreased with the further increase in frequency at the high-frequency domain. This was mainly because the vibration link was a high pass link, which played a major role at the low-frequency domain, while when the electrochemical link was a low-pass link, it played a major role at the high-frequency domain.

Figure 6a shows the sensitivity of three seismic sensors without a compensation circuit or a feedback circuit. The maximum sensitivities and 3dB bandwidth of CME6011, the integrated structure with four planar electrodes, and the integrated structure with four centrosymmetric electrodes were quantified as 371.15 V/(M/s) ≅ 1.4 Hz and 0.33–8.09 Hz, 3555.57 V/(M/s) ≅ 1 Hz and 0.48–3.98 Hz, 15836.16 V/(M/s) ≅ 2 Hz and 0.60–7.10 Hz. According to the experimental results, the maximum sensitivity of the integrated structure with four centrosymmetric electrodes was two orders of magnitude higher than that of CME6011 and three times higher than the integrated structure with four planar electrodes. Compared with the other two seismic sensors, in a full frequency band, the seismic sensor reported in this study was featured with the highest sensitivity, especially at the middle- and high-frequency domains, which were more compatible with the bandwidth extension using a compensation circuit in the later stage.

In terms of the electrode structure, the anode of the integrated structure with four planar electrodes was in a ring shape outside the flow channel, and the cathode was in the center area, which made it difficult for charged ions generated by the anode reaction to reach the central area of the cathode. Especially in high-frequency vibrations, limited durations of ion diffusion led to compromised sensitivities. For the seismic sensor in this study, the ions generated by the anode reaction quickly reacted with the cathodes on the side walls of the channel, avoiding the waste of cathode areas. In addition, the cathodes of the planar four-electrode structure were only distributed on wafer surfaces, while the cathodes of this study were not only distributed on wafer surfaces but also on the side walls of the flow channels, which more effectively captured the charged ions required for the cathode reaction, leading to significant improvements in sensitivities.

Because the anodes of one pair of electrodes and the cathodes of the other pair of electrodes were distributed on the same side of the chip, some of the ions generated by the anode were consumed by the cathodes of the other pair of electrodes, which affected the sensitivity of the device. When low-frequency vibration occurred, the electrolyte flow range was large, and this consumption effect would be more obvious, which should be the reason for the rapid decline of the sensitivity of the geophone in the low-frequency domain.

In order to measure the noise of the device in this study, the seismic sensor developed in this study and the commercial counterpart of CME6011 were placed side by side to monitor voltage noise signal for 600 s in the absence of vibration signals. The collected voltage noise data were transformed from the time domain to the frequency domain through the Fourier transform and divided by the corresponding sensitivity at each frequency point to obtain the noise power spectrum densities, as shown in Figure 6b. In the range of 0.01 Hz to 0.1 Hz, the noise level of the seismic sensor in this study was roughly equal to that of CME6011. In the range of 0.1 to 100 Hz and even wider, the noise level of the seismic sensor in this study was slightly lower than CME6011. More specifically, the quantitative comparisons of the noise spectrum densities between the seismic sensor in this study and CME6011 were as follows: −145.79 db vs. −156.59 db ≅ 0.1 Hz, −182.19 db vs. −173.10 db ≅ 1 Hz, −175.20 db vs. −165.28db ≅ 10 Hz.

In order to characterize the consistency of the proposed sensor, two devices were assembled, and their amplitude frequency response curves were depicted as shown in Figure 6c. The high coincidence degree of the two curves shows that the proposed sensor had high consistency because it adopted the structure of integrating four electrodes on the same chip so that it did not need manual alignment and installation.

**Figure 7:**
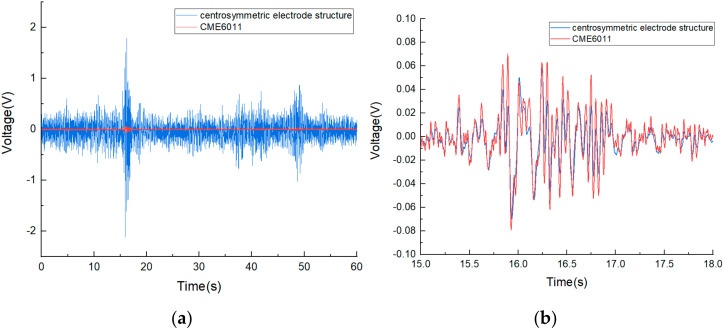
Random vibration response of the electrochemical seismic sensors based on the integrative structure of four centrosymmetric micro electrodes and the commercial counterpart of CME6011 before (**a**) and after (**b**) normalization.

In order to further characterize the performance of the proposed electrochemical seismometer, it was placed side by side with CME6011 to monitor random vibration. Figure 7a shows the random vibration response of electrochemical seismic sensors based on the integrative structure with four centrosymmetric electrodes and the commercial counterpart of CME6011. It can be seen from the figure that since the sensitivity of the seismic sensor developed in this study was higher than CME6011, the outputs of this study were significantly higher than CME6011 under the same random vibrations. After the outputs were normalized, comparable results of the two seismic sensors from 15 s to 18 s were shown in Figure 7b, where high correlations were located, validating the performance of the seismic sensor based on the four centrosymmetric electrodes.

## 6. Conclusions

An electrochemical seismic sensor, based on the integrative structure of four centrosymmetric micro electrodes, was developed in this study. The key parameters of the sensor were determined by numerical simulations, and the sensitivity and noise levels of the sensor were quantified by experimental characterization. Compared with the commercial counterpart of CME6011, the electrochemical seismic sensor reported in this study greatly improved sensitivities by two orders of magnitude. Compared with the previously reported MEMS-based electrochemical seismic sensors based on the integrative structure of four planar electrodes, the seismic sensor in this study also demonstrated about five times higher sensitivity, a simpler fabrication process, and a higher performance at the middle- and high-frequency domains. In future research, the performance of the seismic sensor in this study can be further improved by optimizing the pattern design of the chip surface. The electrochemical seismic sensor in this study may have a broad prospect in resource exploration and seismic monitoring in the future.

## Data Availability

Not applicable.
